# Macrophages and neutrophils in ovarian cancer microenvironment

**DOI:** 10.3389/fimmu.2025.1677441

**Published:** 2025-11-06

**Authors:** Kuang-Chao Cheng, Yu-Hsin Lin, Dao-Sian Wu, Ie-Ming Shih, Tian-Li Wang

**Affiliations:** 1 Department of Gynecology and Obstetrics, Johns Hopkins University School of Medicine, Baltimore, MD, United States; 2 Department of Pathology, Johns Hopkins University School of Medicine, Baltimore, MD, United States; 3 Sidney Kimmel Comprehensive Cancer Center, The Johns Hopkins Medical Institutions, Baltimore, MD, United States

**Keywords:** tumor-associated macrophages (TAMs), tumor-associated neutrophils (TANs), ovarian cancer, tumor microenvironment, immunotherapy

## Abstract

Ovarian cancer (OC) remains one of the most aggressive gynecological malignancies, with a five-year survival rate below 45% despite the recent advances in the introduction of targeted therapy. Moreover, immunotherapy, such as immune checkpoint inhibitors, does not improve the survival of OC patients. Lack of sufficient knowledge in understanding the complexity of the tumor microenvironment likely confers the treatment ineffectiveness. Recently, tumor-associated macrophages (TAMs) and tumor-associated neutrophils (TANs) have garnered research attention as they shape the tumor immune microenvironment, which plays a crucial role in disease progression and treatment response. This article reviews the complex roles of these innate immune cells in OC progression. TAMs represent a significant component of the immune infiltrate in OC, exhibiting considerable functional plasticity and can shift between anti-tumoral (M1) and pro-tumoral (M2) phenotypes. M2-like TAMs typically predominate in the tumor microenvironment, which aids in the development of immune suppression and disease progression. They also contribute to chemoresistance and metastasis; hence, their presence in tumors is associated with a worse prognosis. TANs, like TAMs, exhibit N1/N2 polarization and influence tumor progression through the formation of neutrophil extracellular traps. Understanding the biological interactions between various immune cells and cancer cells may offer new therapeutic opportunities. This review sheds light on the dynamic ecological transformation of the OC tumor microenvironment and highlights the potential of targeting TAM/TAN-mediated processes to improve OC treatment outcomes.

## 1 Introduction

Ovarian cancer (OC) ranks as the eighth most prevalent cancer worldwide, with approximately 313,959 new cases and 207,252 deaths reported each year (1, 2). The five-year cause-specific survival rate for OC varies significantly by stage, ranging from 90% in stage I and 70% in stage II, to 40% in stage III and as low as 20% in stage IV (3). In addition to the lack of early detection methods, late diagnosis often resulted in poor disease outcomes, including resistance to treatment and rapid disease progression. In fact, recurrence occurs in approximately 80% of OC patients (4). Because of the high relapse rates, subsequent treatments tend to be more toxic, significantly impacting patients’ quality of life and incurring substantial financial burdens (5).

**Figure 1:**
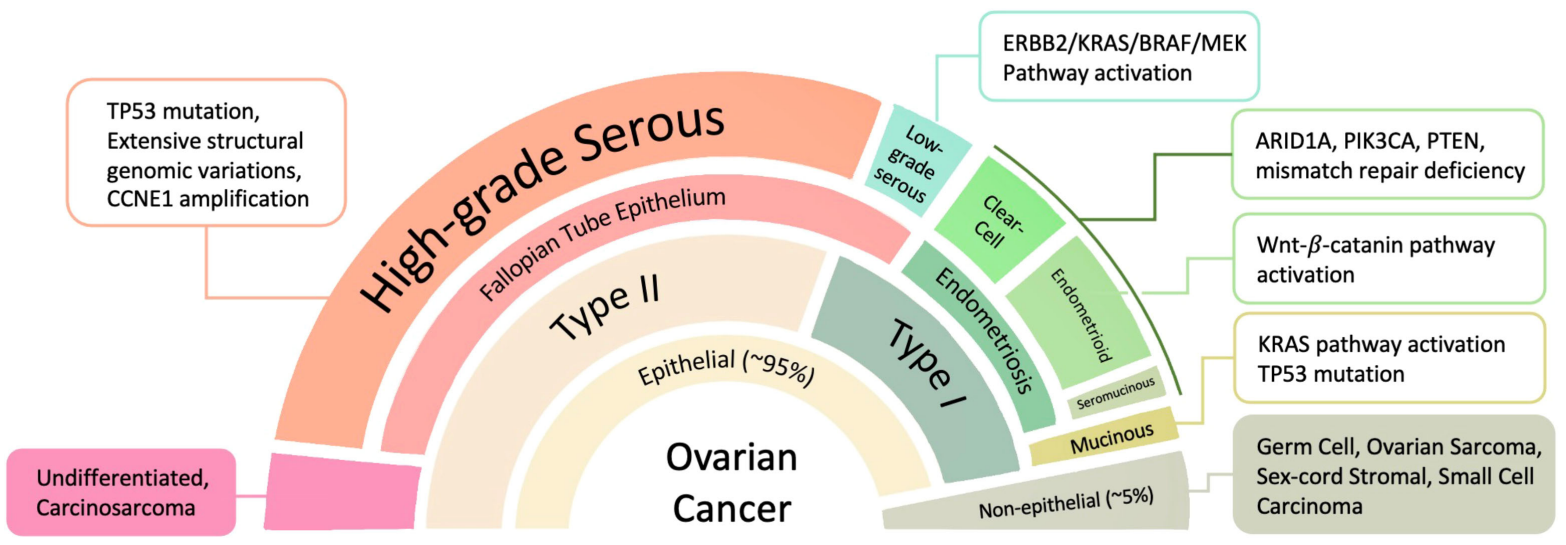
OC subtypes originate from different tissues and feature significant molecular pathway changes. ARID1A, AT-rich interactive domain-containing protein 1A; CCNE1, G1/S-specific cyclin-E1; ErbB, extracellular region binding protein; MEK (alias mitogen-activated protein kinase, MAPK); PIK3CA, phosphatidylinositol 3-kinase catalytic subunit α; PTEN, phosphatase and tensin homologue.

OC displays significant heterogeneity, with diverse histological subtypes originating from different cell types within the ovary. These subtypes vary not only in their morphological and molecular characteristics but also in their behavior, prognosis, and response to treatment. OC can arise from different ovarian tissues, including epithelial, mesenchymal, sex cord stromal, and germ cells. Epithelial tumors account for over 95% of all ovarian malignancies, while stromal and germ cell tumors collectively make up the remaining 5% (6). Among the epithelial tumors, approximately 80% are high-grade serous carcinoma (HGSC), with 75% of these cases diagnosed at FIGO stages III and IV. The remaining 20% includes low-grade serous carcinoma (LGSC), endometrioid, mucinous, clear cell, as well as mixed and undifferentiated carcinomas (7, 8). These OC subtypes can be broadly categorized into two groups based on genetic, molecular, and pathological characteristics (**Figure 1**).

Type I OC includes several distinct histological subtypes: (1) endometrioid, clear cell, and seromucinous carcinomas; (2) low-grade serous carcinomas; and (3) mucinous carcinomas along with malignant Brenner tumors. These malignancies typically originate from benign extraovarian lesions and are characterized by relative genetic stability. They are often diagnosed at early clinical stages and are associated with a comparatively low mortality rate of approximately 10%. Common genetic mutations found in Type I tumors include *PTEN* (phosphatase and tensin homolog), *ERK* (extracellular signal-regulated kinase), *ARID1A* (AT-rich interactive domain-containing protein 1A), *BRAF* (B-Raf proto-oncogene, serine/threonine kinase), *MAPK* (mitogen-activated protein kinase), *PIK3CA* (phosphatidylinositol 4,5-bisphosphate 3-kinase catalytic subunit α), and *KRAS* (Kirsten rat sarcoma viral oncogene homolog) (9).

In contrast, Type II OC is mostly high-grade serous carcinoma, markedly more aggressive, and carries a significantly worse prognosis, primarily due to its tendency to be diagnosed at advanced stages. Type II tumors have high chromosome abnormalities and mutations or DNA copy number variations in key regulatory genes such as *TP53* (tumor protein p53), *RB1* (retinoblastoma 1), *FOXM1* (forkhead box M1), genes encoding *CCNE1* (cyclin E1), and *NOTCH3* (10).

Despite the recent emergence of innovative targeted medications, treatment resistance and the lack of improvement in overall survival rates in OC demand a thorough study of these challenges to develop new strategies. Understanding the immune landscape of OCs represents an emerging research direction. Two of the promising emerging immunotherapeutic approaches are immune checkpoint inhibitors (ICIs) and CAR (Chimeric Antigen Receptor)-T therapy. ICIs work by blocking inhibitory checkpoint ligands on the T cells or tumor cells, effectively lifting the “brakes” on the immune response and reactivating T cell-mediated anti-tumor activity. While ICIs have demonstrated remarkable success in malignancies such as melanoma and endometrial cancer (11), particularly in cases with DNA mismatch repair (MMR) deficiency (12), their efficacy in OC has been limited, with response rates ranging from 10% to 15%. This limited effect is largely attributed to OC’s immunologically “cold” tumor microenvironment, which suppresses effector T cell activation and infiltration (13). Another emerging technique is CAR-T therapy, which provides a precise, individualized approach for each patient by collecting T cells from the patient and re-engineering them to produce CARs on the surface of T cells, which detect cancer cells’ surface antigens and effectively destroy cancer cells. Despite its potential, the use of CAR-T approach faces several obstacles, including difficulty penetrating solid tumor masses, an immunosuppressive tumor microenvironment, and T cell exhaustion (14). Moreover, efforts to identify antigens present on the surfaces of solid tumors but not on healthy cells have largely been unsuccessful.

To overcome these limitations, researchers are exploring other strategies to circumvent the immune-suppressive or “immune-cold” milieu associated with many solid tumors, including OC. The tumor microenvironment (TME) comprises tumor cells, immune cells (such as lymphocytes, dendritic cells, macrophages, and neutrophils), endothelial cells, fibroblasts, and extracellular matrix components, including hyaluronic acid, fibronectin, laminin, and collagen (15). As OC recurs, the tumor undergoes dynamic changes, leading to a more complex and often suppressive immune milieu, which significantly influences treatment outcomes (16).

Given the complexities and evolving nature of the TME, researchers are increasingly focused on understanding the involvement of distinct immune cell populations in disease progression and therapeutic resistance. Among these, tumor-associated macrophages (TAMs) and tumor-associated neutrophils (TANs) have emerged as important regulators of tumor behavior. TAMs and TANs, which are significant components of the innate immune system inside TME, are highly plastic and can adopt diverse phenotypes that either promote or inhibit tumor progression depending on environmental cues (17, 18). In many solid tumors, including OC, TAMs and TANs are often polarized toward pro-tumoral states, contributing to immunosuppression, angiogenesis, metastasis, and therapeutic resistance (19–21). Therapeutic reprogramming of TAMs and TANs is now considered a cutting-edge area of research, with several new medicines entering preclinical and early-phase clinical trials (22, 23). Therefore, this review aims to focus on the emerging and critical roles of TAMs and TANs in OC. We will discuss their origins, phenotypic plasticity, functional heterogeneity, contributions to disease progression, and therapeutic strategies. We seek to highlight the potential of innate immune-targeted therapies to overcome the immune-suppressive obstacles that have hampered the success of traditional immunotherapies in OC.

## 2 The OC TME: origin, composition, and immune landscape

**Figure 2:**
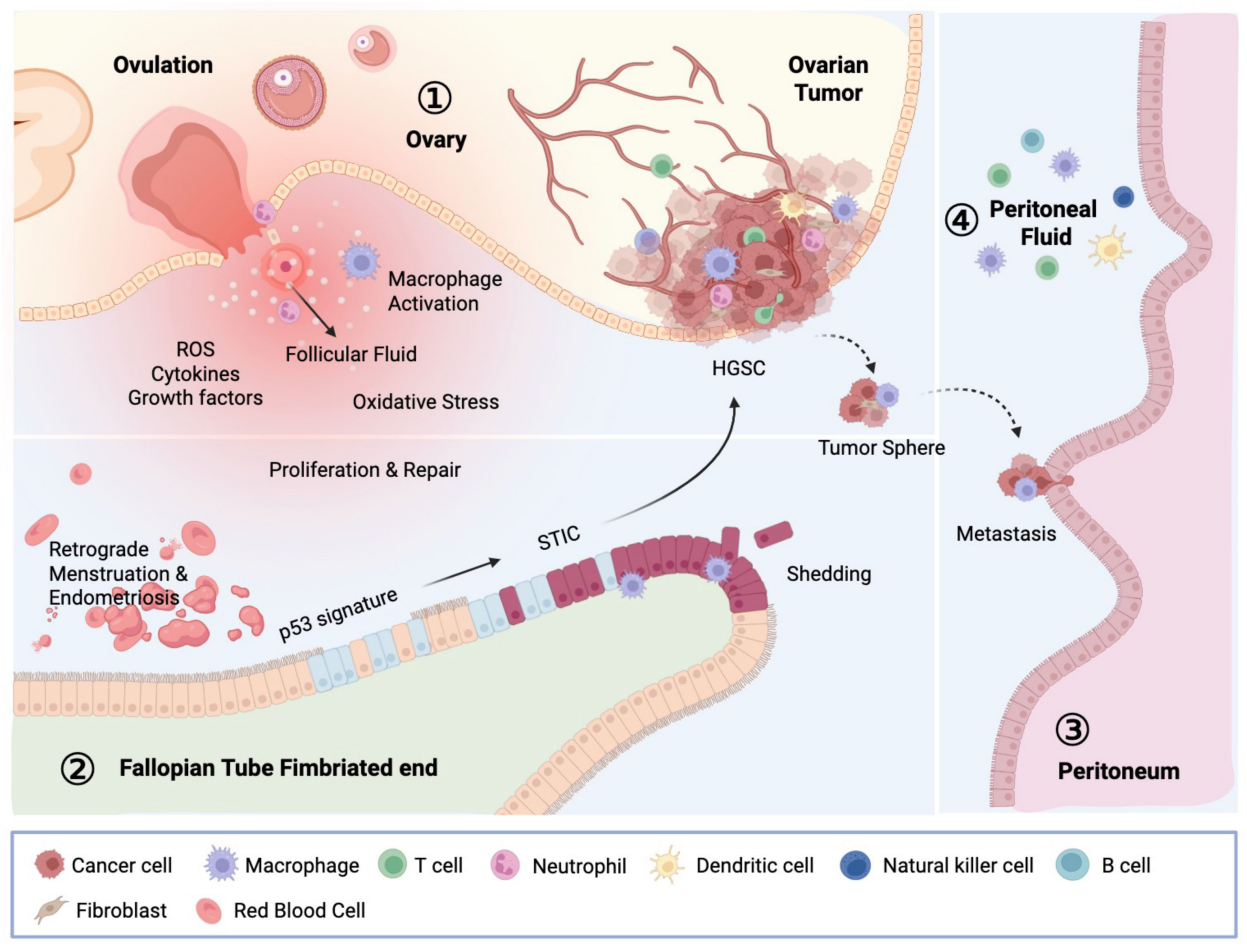
Carcinogenesis of OC and the OC TME. The OC microenvironment comprises the ovary, fallopian tube, peritoneum, and peritoneal fluid, collectively shaping the milieu in which OC develops and progresses. STIC, located at the fimbriated end, is the immediate precursor of HGSC. STIC cells first acquire invasive potential within the fallopian tube, and after detachment, spread across peritoneal surfaces. Those malignant cells encapsulate organs such as the ovary, bowel, peritoneal wall, and omentum. Within the peritoneal cavity, emigrated STIC cells adapt to specific tissue-environmental niches, forming tumor nodules and contributing to the accumulation of tumor ascites. This microenvironment, influenced by ovulation-related damage, infections, and inflammatory conditions, supports tumor progression, metastasis, and the development of chemotherapy resistance. This intricate interplay between tumor cells and immune cells exhibits their crucial role in tumor regulation, therefore affecting patients’ response to therapy. STIC, serous tubal intraepithelial carcinoma; HGSC, high-grade serous carcinoma.

OC, particularly HGSOC, is characterized by a distinct peritoneal TME that coordinates the intricate interactions between tumor cells, resident cells in the peritoneal cavity, and various host immune cells (**Figure 2**). Like many malignancies, OC maintains a chronic inflammatory environment with high amounts of growth hormones, cytokines, chemokines, and reactive oxygen species (ROS), similar to damaged tissues and unhealing wounds (24).

Serous tubal intraepithelial carcinomas (STICs), commonly regarded as precursor lesions of HGSC, are primarily detected in the fimbriae, the distal region of the fallopian tube in close proximity to the ovary (25, 26). The exposure of fallopian tube epithelium, particularly at the fimbriated ends, to follicular fluids during ovulation is hypothesized to be a carcinogenic mechanism that converts fallopian tube epithelial cells to STIC lesions. This is because follicular fluid contains a high concentration of ROS and cytokines, which can directly damage epithelial cell DNA and may cause persistent inflammation. Moreover, tissue damages related to monthly ovulation may also contribute to the inflammatory milieu in the fallopian tubes which add to the malignant alteration of tubal epithelium (24). As a result, incessant ovulation is the highest risk factor of ovarian cancer and reducing ovulation via taking oral contraceptives, surgical removal of ovaries, and pregnancy/breast feeding have been found to reduce OC risks (27).

As the tumor advances, TME becomes increasingly complex and immunosuppressive (28). Innate and adaptive immune cells infiltrate the OC TME and actively shape the tumor immune landscape, which affects treatment response and disease outcome. The TME immune cells include intraepithelial tumor-infiltrating lymphocytes (TILs), natural killer (NK) cells, dendritic cells (DCs), myeloid-derived suppressor cells (MDSCs), tumor-associated macrophages (TAMs), and tumor-associated neutrophils (TANs) (29).

TAMs and TANs are the largest components of innate immune cell populations infiltrating OC. These myeloid-derived cells have garnered increased attention due to their plasticity and significant impact on tumor biology. They not only modulate inflammatory responses and angiogenesis but also influence tumor expansion, invasion, and metastasis. In the following sections, we will provide a comprehensive overview of the functional roles and molecular contributions of TAMs and TANs within the OC microenvironment, emphasizing their potential as therapeutic targets and prognostic markers.

## 3 Macrophages in OC

Macrophages are pivotal components of the innate immune system, possessing phagocytic, antigen-presenting, and hemostatic functions. They protect the host from infection and injury by engulfing and digesting foreign substances and pathogens (30). Upon phagocytizing pathogens, macrophages present antigens via MHC class II molecules to CD4+ T cells, which amplifies the immune response. Furthermore, macrophages play a critical role in tissue repair by recognizing damage-associated molecular patterns (DAMPs) released by tumorigenic cells using toll-like receptors (TLRs) and leads to downstream direct and indirect anti-cancer cellular responds such as T cell activation and TME modification (31).

**Figure 3:**
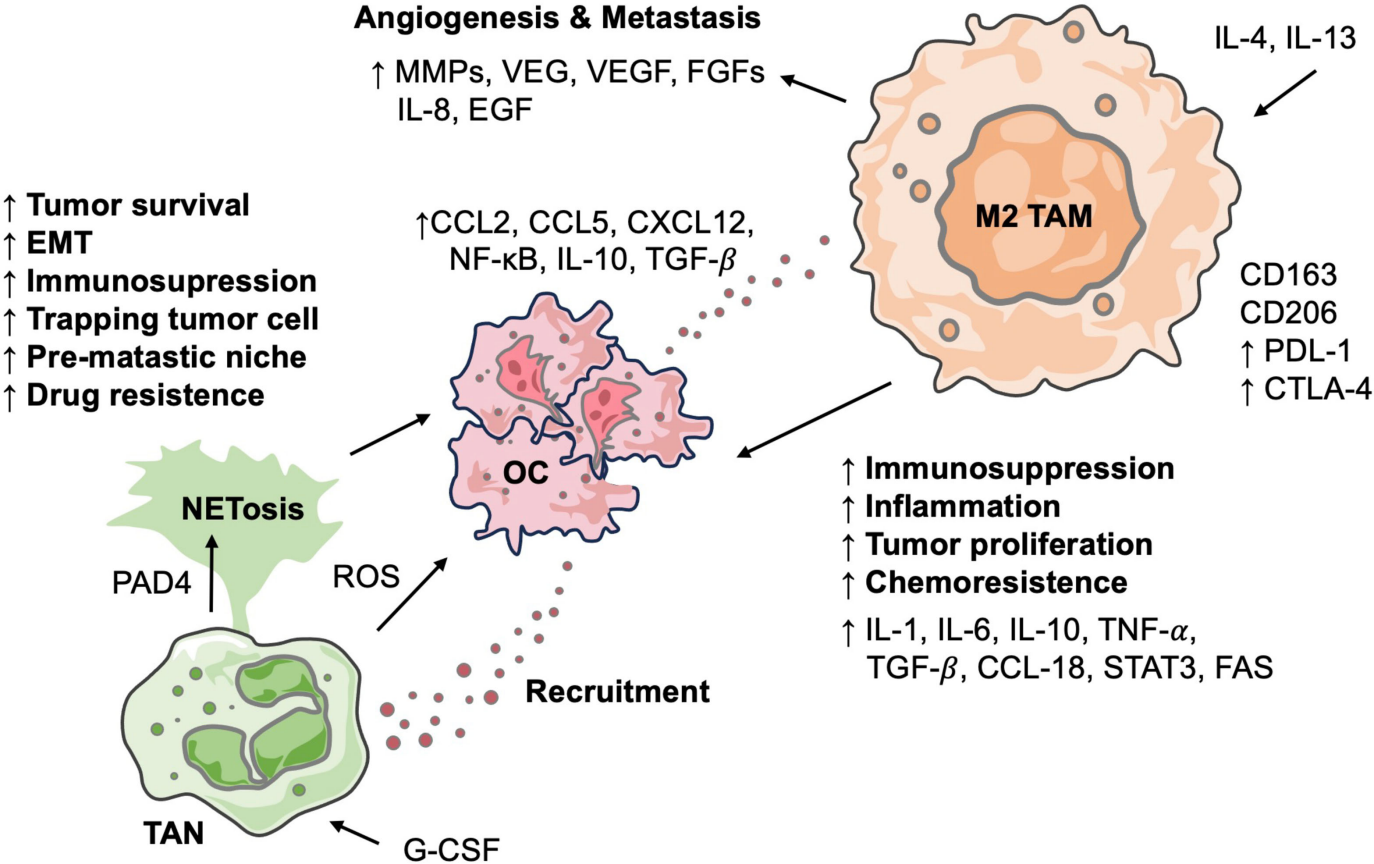
Interactions between neutrophils and macrophages in OC TME. Diagram outlines how TANs and polarized M2 TAMs collaboratively drive OC progression. TANs promote OC progression through NETosis and the release of factors. OC recruit and polarize monocytes into M2 TAMs, which, in turn, are central promoters of angiogenesis, metastasis, inflammation, and chemoresistance by secreting various cytokines. These interactions highlight a crucial immune axis that facilitates tumor malignancy.

Macrophages account for approximately 10% of all hematopoietic cells and represent the most abundant immune population in the OC TME, comprising 39% of immune cells, followed by CD4+ T cells at 12% (32). Their presence in tumor tissues is commonly enriched and is dynamic, heterogeneous, and highly plastic. Depending on their state of polarization, TAMs can exert pro-tumor or anti-tumor actions. Research has demonstrated that TAMs contribute to tumor progression through mechanisms such as angiogenesis, ECM remodeling, metastasis, and the establishment of an immunosuppressive TME, which correlates with poor patient outcomes (33). Moreover, TAMs are critically involved in the development of chemoresistance, significantly impacting the prognosis of cancer patients (**Figure 3**). Recently, TAMs have received considerable attention, and studies in OC have expanded our understanding of their potential as therapeutic targets (**Table 1**).

**Table 1:** Recent advances in targeting TAMs in OC (2025).

Year	Target and mechanism	Study models	Key findings	Clinical implications	Ref
2025	TPL (triptolide), PI3K/AKT/NF-B pathway	*In vitro* & xenograft (mice, 16S rDNA)	TPL inhibits the growth of drug-resistant OC potentially via inhibiting M2 polarization through the PI3K/AKT/NF-kB signaling pathway.	TPL may reverse chemoresistance in OC.	(34)
2025	YTHDF1	Multi-omics bioinformatics, *in vitro*, *in vivo*	YTHDF1 is highly expressed in OC and correlates with poor prognosis. Mechanistically, YTHDF1 is encapsulated within tumor-derived exosomes, promoting the polarization of macrophages toward the immunosuppressive M2a phenotype.	Targeting YTHDF1 could enhance immunotherapeutic responsiveness and improve chemotherapy outcomes by reversing the immunosuppressive microenvironment.	(35)
2025	CYBB	Bioinformatics (multi-database), single-cell sequencing, *in vitro*	CYBB is highly expressed in OC and associated with poor prognosis. It is predominantly expressed in macrophages and its knockout/knockdown suppresses M1 markers while promoting M2 marker expression. CYBB knockdown in TAMs also increased ferroptosis-related proteins (FTH-1, FSP1).	CYBB plays a key role in the immune microenvironment by regulating macrophage infiltration and ferroptosis, positioning it as a potential therapeutic target.	(36)
2025	Rb^high^ M2 TAMs	*In vivo*, ex vivo (human ascites)	Increased Rb expression in TAMs in women with OC is associated with poorer prognosis. The mechanism involves preferential cell death induction in Rb^high^ M2-like immunosuppressive TAMs.	Rb expression in TAMs could serve as a prognostic marker, and the Rb^high^ TAMs are a specific therapeutic target.	(37)
2025	Iron metabolism	Prospective cohort study	HGSOC exhibited an M1-dominant macrophage profile with a high ratio. The ratio positively correlated with inflammatory markers and iron metabolism parameters (hepcidin, ferritin).	The high ratio and associated iron metabolism dysregulation in HGSOC may serve as novel prognostic or functional markers, highlighting the contribution of macrophage plasticity and iron metabolism to disease progression.	(38)
2025	HMOX1	scRNA-seq, bioinformatics (TCGA, GEO, GTEx), *in vitro*, *in vivo*	HMOX1 expression is downregulated in OC epithelial cells but upregulated in macrophages. Both conditions lead to the activation of immunosuppressive macrophage subtypes (SPP1+, FOLR2+ and C1QC+) via the PI3K/AKT/NF-κB (p65) pathway.	HMOX1, TGF-β1, SPP1, FOLR2, and C1QC are confirmed as factors that can be used to construct models predicting the efficacy of immune checkpoint inhibitors.	(39)
2025	CD81, FAK/PCS/Cdh1 pathway	*In vitro*, *in vivo*, Metabolomics, patient tissue	CD81 promotes OC progression by enhancing Bnip3-dependent mitophagy in Tim4+ TAMs via the FAK/PCS/Cdh1 pathway. Stable CD81 knock-down ameliorated disease progression and reversed tumor immunity alterations.	CD81 serves as a prognostic biomarker for poor outcomes. The mechanism of PCS-mediated mitophagy in Tim4+ TAMs deepens the understanding of OC pathogenesis.	(40)
2025	CXCL8-CXCR2 axis, RASGRP4-mediated mTOR-STAT3 pathway	Bioinformatics (TCGA), *in vitro* (THP-1 cells), *in vivo* (xenograft model)	RASGRP4 showed the highest positive correlation with M2 macrophage infiltration among CXCR2 co-expressed genes. The mechanism is: CXCL8-CXCR2 axis enhances M2 polarization through RASGRP4 which activates mTOR-STAT3 signaling.	Higher expression of RASGRP4 is associated with poorer progression-free survival in serous ovarian cancer patients, positioning it as a novel prognostic biomarker and functional regulator of M2 polarization.	(41)
2025	MAFB-WTAP-CD55 axis	Bioinformatics (clinical cohorts, scRNA-seq), preclinical models	MAFB expression is stage-dependently elevated and is a major regulator of OC progression. The -mediated regulation promotes macrophage polarization and regulatory T cell infiltration, leading to immune landscape remodeling and suppression.	The MAFB-WTAP-CD55 axis is identified as a novel and potential therapeutic target to inhibit tumor progression and immune evasion in OC.	(42)
2025	Lactic acid, Gpr132, CD8+ T-cell impairment	*In vitro* (seahorse), *in vivo* (Gpr132-/-mice)	Myc overexpression delays HIF1α degradation in tumor cells and drives metabolic shifts (Warburg effect), resulting in lactic acid secretion. Lactic acid promotes Gpr132-dependent M2 macrophage polarization, which significantly suppresses CD8 T cell function.	Myc overexpression in tumor cells or high lactic acid levels may serve as a prognostic indicator for resistance to immune-based therapies, highlighting the Gpr132 pathway as a critical mechanistic checkpoint.	(43)
2025	Ubiquitin D (UBD), glycolytic reprogramming	Bioinformatics (TCGA, CPTAC), proteomics, gene manipulation, *in vivo*	UBD promotes M2 macrophage polarization through glycolytic reprogramming, which collectively enhances immune evasion and immunotherapy resistance in OC.	Inhibiting UBD or targeting glycolytic pathways may provide new strategies for improving OC immunotherapy efficacy.	(44)
2025	Anlotinib, IL-18	*In vitro*, *in vivo* (orthotopic mouse model)	Anlotinib, a multi-targeted receptor tyrosine kinase inhibitor, promotes M1 macrophage polarization and inhibits M2 polarization by upregulating the secretion of IL-18 from tumor cells.	Anlotinib inhibits OC by converting immunosuppressive TAMs to anti-tumor M1 macrophages via the IL-18 axis.	(45)
2025	Paclitaxel resistance, M2 repolarizer (BMS777607), CSF-1R inhibitor (BLZ945)	Patient-derived organoids, co-culture, humanized patient-derived xenografts	M2 macrophages increase organoid viability and reduce sensitivity to paclitaxel in co-culture. The M2 repolarizer, BMS777607, reduced organoid viability in a macrophage-dependent manner. In a platinum-sensitive huPDX model, the TAM-targeted CSF-1R inhibitor, BLZ945, combined with paclitaxel reduced tumor burden with no regrowth, reversing resistance observed with paclitaxel alone.	Patient-derived organoids and huPDX models are robust platforms for preclinical testing and evaluating immunomodulatory therapy. Targeting TAMs is a viable strategy to overcome paclitaxel resistance in OC.	(46)
2025	Cinobufagin, FOXS1, CCL2/CCR2 pathway	*In vitro*, *in vivo*, transcriptome sequencing	Cinobufagin suppresses Skov3 growth and vasculogenic mimicry (VM) by downregulating FOXS1 and inhibiting IL-4-induced M2 polarization via the CCL2/CCR2 pathway.	Cinobufagin inhibits VM and M2 polarization via FOXS1 and CCL2/CCR2 pathways, showing therapeutic potential in OC.	(47)
2025	P2X7/STAT6 pathway, CAR-T immunotherapy	*In vitro*	P2X7 silencing shifts macrophages toward the M1 phenotype by inhibiting STAT6, reversing M2-mediated CAR-T suppression and enhancing nfP2X7-targeted CAR-T efficacy.	P2X7 targeting reprograms immunosuppressive macrophages and enhances CAR-T efficacy in OC.	(48)
2025	OTUD4, YAP1/CCL2 axis	*In vitro*, *in vivo* (mouse model)	OTUD4 inhibits macrophage recruitment and M2 polarization by blocking YAP1/CCL2 axis, thereby reprogramming TAMs to M1 and suppressing OC metastasis.	Targeting the OTUD4-YAP1-CCL2 axis may inhibit macrophage recruitment and shift TAMs from M2 to anti-tumor M1, offering a therapeutic strategy for OC.	(49)
2025	KLHDC8A, C5a/C5aR/p65 NFκB pathway, C5aR antagonist	Bioinformatics (TCGA), *in vitro*	KLHDC8A knockdown in normal epithelial cells promotes cell proliferation, invasion, migration, and leads to the polarization of pro-tumoral macrophages. This effect is mediated by the C5a/C5aR/p65 NFκB signaling pathway and can be rescued by C5aR antagonists.	KLHDC8A may act as a tumor suppressor gene in OC pathogenesis. Targeting the C5a/C5aR axis using antagonists is a potential therapeutic strategy for OC by modulating macrophage polarization.	(50)
2025	UBE2I, glycolytic reprogramming, PD-L1 expression	*In vitro*, *in vivo* (xenograft mouse model)	UBE2I is upregulated in OC and linked to poor prognosis; its silencing inhibits tumor aggressiveness and drives M1 macrophage polarization via enhanced glycolysis, which in turn promotes PD-L1 expression. Glycolysis inhibitor reversed UBE2I-mediated M1 polarization.	The combinatorial therapy of UBE2I inhibitor plus anti-PD-L1 exhibited higher efficiency than either agent alone, offering a novel avenue to prevent OC progression and enhance immunotherapy.	(51)
2025	TRIM46, CXCL8, Wnt/β-catenin pathway, CXCR1/2 inhibitor (Reparixin)	*In vitro*, bioinformatics (GSEA), patient data (ascites MQs)	TRIM46, upregulated in OC by TAM-derived CXCL8, drives invasion and EMT via the Wnt/β-catenin pathway, contributing to poor prognosis; CXCR1/2 inhibition blocks this effect.	TRIM46 is a prognostic biomarker and a key mediator of TAM-induced invasion. Targeting the CXCL8/CXCR1/2 axis is a potential therapeutic strategy to suppress TRIM46 expression and inhibit OC metastasis.	(52)
2025	Tumor exosomal miR-205, PTEN, PI3K/Akt/mTOR pathway	*In vitro*, *in vivo*, patient data	High miR-205 in OC promotes M2 macrophage polarization via PTEN/PI3K/AKT/mTOR pathway, enhancing tumor progression and poor prognosis.	Exosomal miR-205 shapes the OC microenvironment and is a potential target for therapies disrupting tumor-immune interactions.	(53)
2025	TAM-derived exosomal miR-589-3p, BCL2L13	*In vitro*	TAM-derived exosomes promote OC cell proliferation and inhibit apoptosis via miR-589-3p, which targets BCL2L13; blocking miR-589-3p in exosomes reduces these effects.	The TAM exosomal miR-589-3p/BCL2L13 axis is a potential therapeutic target to inhibit OC proliferation and induce apoptosis.	(54)
2025	Nanovaccine (PLGA-CpG@ID8-M), Gbp2-Pin1-NFκB pathway	*In vitro*, *in vivo*, transcriptome sequencing, proteomics	The PLGA-CpG@ID8-M nanovaccine overcomes free CpG accumulation, reprograms TAMs to tumoricidal M1 macrophages via Gbp2/Pin1–NFκB signaling, inhibits tumor growth, and counteracts chemotherapy-induced immunosuppression by boosting M1 TAMs and lowering tumor CD47.	This study presents a nanovaccine strategy targeting the Gbp2-Pin1-NFκB pathway to remodel TAMs, synergize with chemotherapy, and improve OC outcomes.	(55)
2025	CD163+ macrophages, CD47 blockade, phagocytosis checkpoint LILRB1	*In vitro* (A2780 OC cells)	Tumor conditioning upregulates macrophage CD163, CD206, CD80, and LILRB1 without impairing phagocytosis; CD47 blockade similarly enhances A2780 cell clearance in conditioned and control macrophages.	CD163+ macrophages remain responsive to CD47 blockade, making them promising immunotherapy targets in OC despite tumor-induced immunosuppression.	(56)
2025	GNA15	Bioinformatics (RNAseq, TCGA, Cox regression, LASSO regression, GSEA), *in vitro*	GNA15 is upregulated in cisplatin-resistant OC, promotes proliferation, correlates with M2-like TAM infiltration and CD163, and participates in immune processes; an eight-gene TAM-related model including GNA15 predicts poor prognosis.	GNA15 is a potential prognostic marker and therapeutic target in OC, associated with M2-like TAM polarization and cisplatin resistance.	(57)
2025	IL-33, TRIM28, PI3K/Akt pathway, glycolysis	RNA-Seq, ChIP-Seq, *in vitro*	IL-33 interacts with TRIM28 to activate PI3K/Akt–mediated glycolysis in BMDMs, suppressing M2 polarization and inhibiting OC growth independently of ST2.	Targeting the ST2-independent IL-33/TRIM28 axis to block M2 polarization and macrophage glycolysis offers a potential OC therapy.	(58)
2025	CD44-targeted docetaxel (DTX)-loaded nanoparticles (CD44-PLGA-DTX NPs)	*In vitro*, cytokine profiling	CD44-PLGA-DTX NPs enhance spheroid uptake, reduce cell viability, reverse chemoresistance, and reprogram TAMs from M2 to M1, modeling CSC- and TAM-driven chemoresistance.	CD44-targeted PLGA-DTX NPs represent a dual-targeting therapeutic strategy, overcoming both cancer stem cell-driven chemoresistance and TAM-induced immunosuppression.	(59)
2025	ACTN1, FBXO25, ERK1/2 signaling	Bioinformatics (HPA, TCGA, Kaplan-Meier Plotter, TIMER2.0), *in vitro*	ACTN1, upregulated in OC and linked to poor prognosis, promotes tumor growth, EMT, and M2 macrophage polarization via ERK1/2 signaling; FBXO25 interacts upstream, and ERK1/2 inhibition partially reverses these effects.	The FBXO25/ACTN1/ERK1/2 axis and M2 macrophages may represent promising targets for developing OC treatments.	(60)
2025	Curcumin	*In vitro*	High-dose Curcumin inhibits cancer proliferation, while low-dose suppresses TAM-induced malignancy by reducing M2 polarization, modulating cytokines, and limiting migration, invasion, and EMT.	Low-dose curcumin exerts anti-tumor effects by modulating TAMs, offering potential new strategies for OC treatment.	(61)
2025	MOv18 IgE antibody (anti-folate receptor-α)	Ex vivo, high-dimensional flow cytometry, RNA-seq, clinical phase I trial data	Patient-derived macrophages are immunosuppressive and FcϵR+, promoting Treg cells. MOv18 IgE reprograms them to a pro-inflammatory, T cell-stimulatory state, reducing Treg induction, boosting CD8+ T cells, and generating an IgE-driven immune signature in tumors.	MOv18 IgE therapy repolarizes macrophages to a hyperinflammatory state, suppressing Tregs and enhancing anti-tumor immune activation.	(62)
2025	ACSL4, USP7, ferroptosis	*In vitro*	ACSL4, low in EOC, suppresses tumor growth by inducing ferroptosis and M1 macrophage polarization, counteracting USP7-driven antiferroptosis and M1 suppression.	ACSL4 suppresses EOC growth and survival by counteracting USP7-driven antiferroptosis and M1 macrophage inhibition, identifying this pathway as a potential therapeutic target.	(63)
2025	Gallic acid, PI3K-AKT pathway	*In vitro*, *in vivo*	Gallic acid inhibits OC growth and metastasis by suppressing PI3K-AKT signaling, enhancing macrophage cytotoxicity, and promoting M1 polarization in the ID8 tumor microenvironment.	Gallic acid plays an anticancer effect via blockage of the PI3K-AKT pathway.	(64)
2025	PTTG1, cGMP-PKG pathway	Bioinformatics (GSE135886), *in vitro*	PTTG1 drives EOC proliferation, invasion, and EMT by activating the cGMP-PKG pathway, inducing M2 macrophage polarization; knockdown reverses these effects.	This study uncovers a novel mechanism of PTTG1 in OC development and suggests it as a potential therapeutic target.	(65)
2025	CXCL11, JAK2/STAT1 pathway	Bioinformatics, *in vitro*	CXCL11, a protective biomarker, promotes M1 macrophage polarization via JAK2/STAT1, supporting the anti-tumor role of M1 TAMs in OC.	CXCL11 emerges as a potential therapeutic target and prognostic marker, providing new avenues for OC immunotherapy.	(66)
2025	SNX10, mTOR1/Lysosomes pathway	Bioinformatics (scRNA-seq, Kaplan-Meier Plotter, GEPIA2), *in vitro*	SNX10 in TAMs drives M2 polarization, enhancing OC migration, invasion, and cisplatin resistance by reducing lipid droplets, inhibiting p-mTOR1, and impairing lysosomal function, while modulating PD-L1 expression based on platinum sensitivity.	SNX10 regulates TAMs through the mTOR1/lysosome pathway, affecting lipid metabolism and PD-L1, and represents a potential target to counter metastasis and chemoresistance in OC.	(67)
2025	Exosomal CMTM4	*In vitro*, *in vivo*, patient data (prognostic association)	Tumor-derived exosomal CMTM4 induces M2 macrophage polarization and immune suppression via NF-κB-mediated cytokine and ICAM1 upregulation, promoting metastasis; CMTM4 depletion enhances anti-PD-1 sensitivity.	Eltrombopag inhibits CMTM4, enhancing PD-1 immunotherapy, while the exosomal CMTM4–ICAM1–CD206 axis serves as a prognostic marker and therapeutic target in OC.	(68)
2025	RelA (p65), Pol η/TLS pathway	*In vitro*, *in vivo*	TAMs enhance cisplatin resistance in OC by upregulating TLS pathway proteins (Pol η, RAD18, REV1) and downregulating NER, with RelA (p65) recruiting Pol η; pristimerin disrupts RelA translocation, impairing DNA repair and promoting cell death.	The TAM–RelA–Pol η/TLS axis drives cisplatin resistance, and RelA inhibition (e.g., pristimerin) may sensitize OC cells to platinum therapy.	(69)

### 3.1 The M1/M2 dichotomy and spectrum of TAMs

The heterogeneous population of TAMs has been broadly divided into the M1/M2 dichotomy based on their metabolic profiles, immunological responses, and activation states (70). Traditionally, M1 phenotype macrophages, also known as classically activated macrophages, exhibit anti-tumorigenic behavior by producing angiostatic factors, such as IL-12, IL-23, and CXCL10, when activated by bacterial products like lipopolysaccharide and pro-inflammatory cytokines (71). Due to the accumulation of Kreb cycle metabolites, these macrophages exhibit enhanced antigen-presenting capabilities, marked by increased expression of MHC class II, CD80, and CD86, and elevated production of NO, reactive oxygen intermediates (ROIs), and prostaglandins, collectively reinforcing their pro-inflammatory phenotype (72, 73).

In contrast, M2 phenotype macrophages, also known as alternatively activated macrophages, are pro-tumorigenic cells stimulated by Th2-related cytokines, including IL-4, IL-10, IL-13, and TGF-β. This stimulation leads to increased expression of dectin-1, C-type lectin DC-SIGN, mannose receptor, scavenger receptor A (SR-A), scavenger receptor B-1 (SR-B1), CD163, CD68, CCR2, CXCR1, CXCR2, VEGF-A, and MGL-1 (70). Furthermore, T-cells finely tune macrophage polarization via the CD40-CD40L interaction, where specific ligand residues encode distinct messages (74). Unlike M1 macrophages, M2 macrophages utilize arginine metabolism for ornithine production and generate substrates for fatty acid oxidation (FAO), a critical energy source (75). Elevated serum ornithine levels have been found in many cancer patients. In addition to serving as an energy source, lipid metabolism plays a crucial role in TAM functionality. Studies have demonstrated that FAO is critical for maintaining an immunosuppressive TME and modulating the antigen-presenting capacity of immune cells (76–78). M2 macrophages are implicated in promoting tumor growth, facilitating invasion and metastasis, and fostering an immunosuppressive TME. A low M1/M2 ratio is correlated with poor prognosis, while a high M1/M2 ratio indicates the opposite; this phenomenon is observed in many cancers, including OC (79).

Of all the TAMs in OC, >50% exhibit an M2 phenotype, while M0 and M1 phenotypes account for the remaining populations (80). However, recent studies challenge the classical M1/M2 dichotomy, the TAM population consists of a spectrum of phenotypes with overlapping functions (81). With technology advances, we are now discovering TAMs that do not fit into current categories, and TAM subtypes can differ significantly cancer type, stage, and histological landscape, necessitating more investigation and characterization (82). Some researchers classified the more complex M2 phenotype into 4 subtypes: M2a [alternatively activated, M(IL-4)], M2b (Type 2 macrophages, M(Ic)), M2c (Deactivated macrophages, further separated into M[IL-10), M(GC), and M(GC+TGFβ)], and M2d (83). Beyond this, single-cell RNA sequencing has been employed to investigate cellular diversity in several malignancies, and up to seven subtypes of TAMs have been identified based on expressed genes, pathways, and functions (84, 85). Even so, the M1/M2 structure is still widely used because of the extensive experimental data accumulated.

### 3.2 TAMs in OC progression

TAMs represent a major component of the TME in OC and play a critical role in disease progression. They often skewed toward an M2-like phenotype, contributing to multiple oncogenic processes through complex interactions with cancer cells and stromal elements. Understanding the multifaceted roles of TAMs in the progression of OC is essential for identifying new therapeutic targets and improving patient outcomes.

#### 3.2.1 Chemoresistance

TAMs significantly contribute to chemoresistance in OC through mechanisms involving immune modulation, cytokine secretion, and metabolic reprogramming.

Co-culture studies have demonstrated that interactions between TAMs and OC cells lead to the upregulation of PD-L1 in both cell types (86). This upregulation is associated with increased expression of IL-6, IL-10, VEGF, STAT3, B-cell lymphoma 2 (BCL2), and multidrug resistance protein 1 (MDR1) in OC cells, thereby promoting proliferation, migration, and resistance to carboplatin (86). Crucially, silencing PD-L1 restores carboplatin sensitivity. Recently, this PD-L1-mediated resistance is supported by several molecular pathways that promote M2 polarization. For example, UBE2I upregulation in OC drives M1 macrophage polarization via enhanced glycolysis, which promotes PD-L1 expression, with UBE2I inhibitors synergizing with anti-PD-L1 therapy to enhance efficacy (51). Similarly, the TAM protein SNX10 drives M2 polarization, enhances cisplatin resistance, and modulates PD-L1 expression by inhibiting the mTOR1/lysosome pathway and disrupting lipid metabolism, presenting a target for anti-metastasis and chemosensitization (67). Furthermore, tumor-derived exosomal CMTM4 induces M2 macrophage polarization and immune suppression via the NF-B pathway and ICAM1 upregulation, promoting metastasis and attenuating anti-PD-1 sensitivity; its inhibition with Eltrombopag can enhance immunotherapy (68).

The CCL2/CCR2 axis recruits monocytes to the OC TME, where CCL2 drives their M2-like TAM differentiation, promoting tumor growth and chemoresistance. Paclitaxel-resistant OC cells drive chemoresistance by secreting CCL2, which recruits and polarizes macrophages into M2-like TAMs that reinforce resistance by secreting IL-6 and IL-10; consequently, inhibition of the CCL2/CCR2 signaling pathway restores paclitaxel sensitivity (87). Recent studies shows, cinobufungin inhibited IL-4 induced M2 polarization via the CCL2/CCR2 pathway (47), and OTUD4 inhibits macrophage recruitment and M2 polarization by blocking Y.

AP1/CCL2 axis (49). Furthermore, UBR5 (Ubiquitin protein ligase E3 component n-recognin 5) is implicated in recruiting TAMs to OC cells via the CCL20-CCR6 axis (88), potentially contributing to increased metastasis and paclitaxel resistance (89). Compounding this, platinum treatment may activate the STAT3 pathway by increasing IL-6, IL-10, and PGE2 production, leading to M2 polarization and tumor progression (90).

Metabolic reprogramming of TAMs is another mechanism contributing to chemoresistance. Gaude et al. (2018) identified metabolic heterogeneity in OC, defining low- and high-OXPHOS subtypes. High-OXPHOS tumors depend on the PML–PGC-1α axis to sustain oxidative metabolism and exhibit enhanced chemosensitivity driven by oxidative stress and ferroptosis (91). Additionally, enhanced fatty acid (FA) uptake and metabolism are another key feature of metabolic reprogramming in OC. The primary tumor and omental metastatic sites are enriched in FAs due to ascitic fluid accumulation and adipocyte-derived secretions (92). Moreover, OC induce cholesterol efflux from TAMs through ATP-binding cassette (ABC) transporters, depleting of lipid rafts and promoting IL-4-mediated M2 polarization. This reprogramming suppresses IFN-γ-induced gene expression, facilitating an immunosuppressive TME. Genetic deletion of ABC transporters in TAMs reverses these effects (93).

#### 3.2.2 Immunosuppression and inflammation

TAMs play a pivotal role in establishing an immunosuppressive TME, facilitating tumor progression and immune evasion. TAMs secrete immunosuppressive cytokines such as IL-10 and TGF-β, which inhibit the function of effector T cells and NK cells, thereby dampening anti-tumor immune responses. Specifically, TGF-β impairs mitochondrial respiration in CD4+ T cells, leading to reduced production of IFN-γ and granzyme B, crucial components of cytotoxic activity (94, 95). The immunosuppressive milieu orchestrated by TAMs is further compounded by their interactions with other immune cells and factors within the TME. TAMs have been implicated in the suppression of dendritic cell maturation and function, as well as the inhibition of NK cell cytotoxicity. Moreover, the expression of immune checkpoint molecules such as PD-L1 and CTLA-4 on TAMs contributes to the attenuation of T cell activation and proliferation (96).

In addition to their immunosuppressive functions, TAMs promote an inflammatory environment that promotes tumor progression. They secrete pro-inflammatory cytokines and chemokines, including TNF-α, IL-1β, and CCL18, which facilitate tumor cell proliferation, angiogenesis, and metastasis. The dual role of TAMs in mediating immunosuppression and inflammation underscores their significance in the pathophysiology of OC and highlights the potential of targeting TAMs as a therapeutic strategy (32).

#### 3.2.3 Angiogenesis and metastasis

Angiogenesis is essential for tumor growth beyond a certain size, as the expanded vasculature supplies oxygen and nutrients while providing routes for metastasis. M2 TAMs are key derivers of this process, secreting pro-angiogenic factors such as matrix metalloproteinases (MMPs), fibroblast growth factors (FGFs), and vascular endothelial growth factor (VEGF). Affymetrix gene profiling of TAMs isolated from OC expressed genes associated with extracellular matrix remodeling, including high levels of cathepsins (L, C, Z, and B), urokinase-type plasminogen activator (uPA), lysosomal enzymes, ADAM proteases, and MMPs (1, 9, 12, and 14), which facilitate ECM degradation and enable vessel sprouting and remodeling (97). TAM-derived MMPs remodel the ECM, promoting endothelial cell migration during angiogenesis, while FGFs and cytokines further stimulate endothelial proliferation and blood vessel formation (98). Additionally, TIE2+ TAMs are abundant in OC lesions, ascites, and patient’s blood, correlating positively with microvessel density (99). Ang2, the TIE2 ligand, promotes TIE2+ TAM recruitment to the TME, enhancing angiogenesis through IGF-1 signaling (99).

VEGF, produced by TAMs, binds to endothelial cell receptors and stimulates the formation of new blood vessels to supply the tumor with nutrients and oxygen, hence boosting tumor growth and survival. Preclinical studies have shown that overexpression of VEGF can transform normal ovarian epithelium into ascites-producing, neoplastic tissue (100). Additionally, VEGF may suppress T cell activation and proliferation, contributing to immune evasion (101). High levels of VEGF have been observed in both primary OC and ascitic fluid, and their expression is strongly correlated with poor patient survival (102, 103). Pre-operative plasma VEGF-C levels were highly associated with recurrence and poor prognosis in OC patients (104). Additionally, VEGF expression is higher in OC-induced ascites than in ascitic fluids of nonmalignant origin (105). Recent evidence from Zhou et al. demonstrates that VEGF/VEGFR inhibitors significantly improve progression-free and overall survival in patients with platinum-resistant OC, while maintaining a manageable safety profile (106). Notably, macrophage depletion alone has been shown to reduce VEGF levels, thereby limiting the accumulation of ascites and metastatic dissemination (107).

TAMs play a critical role in facilitating tumor metastasis. In the peritoneal cavity, TAMs contribute to the formation of multicellular spheroids with tumor cells, providing a protective environment that enhances tumor survival and facilitates peritoneal dissemination. Also, studies reveal that M2 macrophage–derived CCL4 activates the CCR5/PI3K pathway in mesothelial cells, inducing P-selectin expression. This facilitates CD24-mediated tumor–mesothelial adhesion *in vitro* and *in vivo*(108). TAMs within spheroids can secrete epidermal growth factor (EGF), resulting in the downstream upregulation of EGFR and VEGF signaling that promote tumor cell proliferation and migration. In mouse OC models treated with erlotinib, an EGFR inhibitor, exhibited reduced spheroid formation and metastatic progression, underscoring the important role of TAMs in disease progression (32). Moreover, EGF upregulates αMβ2 integrin on TAMs and ICAM-1 on tumor cells. Therefore, blocking EGFR signaling or neutralizing ICAM-1 reduced spheroid formation and cancer progression in mouse models (109, 110).

TAMs also play a pivotal role in promoting tumor cell dissemination by inducing epithelial-mesenchymal transition (EMT)—a process in which epithelial cells acquire mesenchymal traits, thereby enhancing their migratory and invasive capabilities. TAM-derived cytokines such as IL-6 and TNF-α activate signaling pathways like STAT3 and NF-κB in tumor cells, inducing EMT and increasing metastatic potential (111). Building on these findings, Li et al. demonstrated that TAM-derived CXCL8 promotes OC cell invasion by upregulating TRIM46 expression, which activates the Wnt/β-catenin signaling pathway and induces EMT (52). Furthermore, TAMs secrete MMPs that degrade the extracellular matrix, allowing tumor cells to invade surrounding tissues and enter the circulation (112).

## 4 Neutrophil in OC

Neutrophils are the most abundant circulating leukocytes and play a critical role in bridging innate and adaptive immunity. They are among the first immune cells recruited to areas of inflammation or malignancies and can influence the activity of other immune cells, including those of the adaptive immune system. Neutrophils have a short lifespan of approximately 7–10 hours in both humans and mice. However, cytokines secreted by tumor cells, such as G-CSF, IL-1β, IL-6, and TNF, can extend their longevity (113–115). Indeed, neutrophils are now recognized to be much longer-lived than previously thought, surviving for 5 days or more in the circulation (116), and they may even be able to survive for weeks in tissues.

In various malignancies—including lung, breast, and gastric cancers—neutrophils constitute a substantial portion of immune cells infiltrating primary tumors, and their presence has been consistently associated with reduced overall survival and recurrence-free survival (117, 118). Extensive evidence supports a pro-tumor role for neutrophils in cancer progression. For instance, Bekes et al. demonstrated that neutrophils produce MMP9 within the TME, promoting angiogenesis, tumor growth, and metastasis in mouse transplantation models (119). Similarly, Yang et al. reported that elevated infiltration of TANs in epithelial OC impairs CD8+ T cell cytotoxicity, thereby fostering an immune-tolerant microenvironment and increasing the risk of recurrence (120). In HGSOC, TANs have also been shown to express high levels of immunosuppressive markers such as PD-L1 and CD14, with their presence correlating with diminished T cell function (121). Despite the growing recognition of NETs in cancer biology, their roles in OC have only begun to be explored (**Table 2**). The following subsections provide a detailed description of TANs.

**Table 2:** Current studies to TANs in OC (Last 5 years).

Year	Target and Mechanism	Study Models	Key Findings	Clinical Implications	Ref
2025	NETosis-related genes	Bioinformatics (TCGA-OV, GTEx, H&E digital slides)	Four NETosis-related genes were identified as prognostic in OSC. The LR pathomic model stratified patients into prognostic groups with strong classification performance (AUC = 0.761).	NETosis genes serve as non-invasive prognostic biomarkers in OSC.	(122)
2025	NETs, IL-10+ B cell	*In vitro*, *in vivo*	NETs formed in omental fat-associated lymphoid clusters induce CXCL13 in mesothelial cells, recruiting IL-10–producing innate-like B cells that expand Tregs and promote omental metastasis.	NETs drive immunosuppressive pre-metastatic niche formation via IL-10+ B cell expansion.	(123)
2025	NETs, extracellular DNA, and complement activation (C3b/c)	Clinical prospective study	High serum genomic DNA, MPO, CitH3, and C3b/c correlated with poor OS, while ascites factor H predicted improved outcomes. Combined low C3b/c and low CitH3 or MPO identified patients with significantly better survival.	NET formation, extracellular DNA, and complement activation serve prognostic biomarkers in advanced EOC.	(124)
2025	JAG2+ TANs, Notch1/RBPJ pathway	Bioinformatics (scRNA-seq, TCGA meta-analysis), *in vivo*, *in vitro*, ex vivo	JAG2+ TANs induced effector Treg differentiation via Notch1/RBPJ signaling, promoting IL-10+, ICOS+, CTLA4+, CD103+ Tregs and driving PD-1 resistance.	JAG2+ TANs act as biomarkers of immune evasion and PD-1 blockade resistance; Notch or JAG2 inhibition reprograms Tregs and sensitizes tumors to immunotherapy in HGSC.	(121)
2025	NETs-related lncRNAs (e.g., GAS5)	Bioinformatics (TCGA-OV, GTEx, Harbin validation), *in vitro*, *in vivo*	Six NETs-related lncRNAs formed a prognostic model distinguishing high- and low-risk patients. GAS5 knockdown reduced invasion, while overexpression enhanced malignancy. NETs induction *in vivo* increased CitH3 and abdominal metastasis.	The NETs-lncRNA signature predicts prognosis and therapy response in OC.	(125)
2024	G-CSF–induced NET formation, PAD4-dependent peritoneal dissemination	*In vitro*, *in vivo*	Neutrophilia correlated with advanced peritoneal spread, higher ascitic neutrophils, elevated G-CSF, and abundant NET foci, all reversed by PAD4 inhibition.	NETs act as drivers of peritoneal dissemination.	(126)
2024	NETs-related gene signature (RAC2, SELL), immune landscape remodeling and drug response prediction	Bioinformatics (TCGA-OV, ICGC-OV, GTEx, single-cell RNA-seq, pan-cancer datasets)	An 8-gene NETs signature classified OC patients by prognosis and therapy sensitivity. High-risk tumors had elevated RAC2, more neutrophils/Tfh cells, and reduced M1 macrophages, correlating with immunosuppression and poor OS. SELL expression predicted favorable outcomes.	NETs-related genes, particularly RAC2, serve as prognostic and therapeutic biomarkers.	(127)
2023	NETosis biomarkers	Biomarker analysis	NETosis markers were elevated in tumor and ascitic environments of HGSOC, promoting disease progression. Neoadjuvant therapy reduced systemic but not local NET biomarkers.	NETosis is active in advanced HGSOC; plasma cfDNA and calprotectin may serve as biomarkers.	(128)
2022	Circulating NET markers	Clinical plasma analysis	Circulating NET markers were not elevated in OC patients, showing no correlation with survival or disease progression.	Findings contradict prior reports suggesting cfDNA and calprotectin as NET-derived biomarkers.	(129)
2022	NETs, physical barrier reducing doxorubicin diffusion	*In vitro*, ex vivo	NETs impeded doxorubicin diffusion, diminishing its cytotoxicity toward OC cells. DNase I–mediated NET degradation restored drug penetration and efficacy.	NETs hinder chemotherapy delivery in OC; enzymatic NET degradation may enhance doxorubicin responsiveness.	(130)
2020	NETosis, S100A8/CRP ratio	*In vitro*	NETosis influenced the tumor environment, correlating with non-miliary metastasis and improved OS. The S100A8/CRP ratio associated with better survival outcomes.	Findings indicate a context-dependent dual role of NETs/TANs in OC—potentially pro-metastatic or immune-supportive depending on tumor microenvironment context.	(131)

### 4.1 TANs polarization

Based on animal studies, in 2009 Fridlender et al. proposed a hypothesis that TANs, like TAMs, can be polarized into anti-tumor (N1 type) and pro-tumor (N2 type) phenotypes (132). However, it is largely unknown whether the N1/N2 profile observed in mouse models applies to human TANs. Both polarization pathways are orchestrated by cytokines within the TME (133). This polarization is reversible; for example, blocking TGF-β can repolarize TANs from the N2 state back to the N1 state (132).

N1 TANs exhibit anti-tumor properties, including enhanced production of immunostimulatory cytokines and chemokines, reduced expression of arginase, and increased cytotoxicity against tumor cells *in vitro*. Moreover, neutrophil-derived oxidants, cytokines, and enzymes contribute to tumor suppression. For example, ROS generated by neutrophils activate an H_2_O_2_-dependent calcium channel in cancer cells, leading to calcium influx and subsequent cell death (134, 135). Furthermore, neutrophils have the capacity to produce TNF-related apoptosis-inducing ligand (TRAIL), which induces apoptosis in cancer cells. The efficacy of this pathway is further enhanced by stimulating neutrophils with IFN-γ (136). In contrast, neutrophils with an N2-like phenotype promote invasion and metastasis in OC by upregulating MAPK signaling (137). Upregulation of ARG1 is also associated with N2’s tumor-supportive, T cell inhibitory phenotype (138).

Despite these findings, the precise phenotypic classification of TANs remains controversial. While it is well-established that neutrophils express diverse surface markers and receptors that may influence tumor progression and clinical outcomes, the existence and functional relevance of distinct pro-tumor and anti-tumor TAN subsets in human cancers require further investigation.

### 4.2 Tumor-induced NETosis

The intricate connection between OC development and neutrophil extracellular traps (NETs) has been the subject of recent research. NETs formation was first recognized as a mechanism by which neutrophils ensnare and destroy microorganisms (139). NETs, released by neutrophils in response to external pathogens, are primarily composed of fibrous decondensed chromatin bound with histones, myeloperoxidase (MPO), and various cytoplasmic proteins such as neutrophil elastase, cathepsin G, and lactoferrin (140, 141).

NET release, also known as ‘NETosis’, was identified in biopsy samples from two out of eight pediatric patients with Ewing sarcoma (142). In the context of cancer, NETs have been implicated in promoting thrombosis, systemic inflammation, and multi-organ failure (143). NETs also play a role in tumor survival, pre-metastatic niche development, and resistance to treatments (144). In OC, neutrophils are drawn to the omental niche by tumor-derived cytokines such as IL-8, growth-regulated oncogenes α/β (GROα/β), G-CSF, and monocyte chemoattractant protein-1 (MCP-1) (145). Infiltrating neutrophils can produce NETs, resulting in a pro-metastatic milieu that favors tumor implantation and progression, a phenomenon called “neutrophil-assisted soil preparation in metastasis” (145).

Supporting these findings, Singel et al. (2019) demonstrated that ascitic fluid from OC patients chemoattracted neutrophils and induced NET release *in vitro*, an effect attenuated by DNase treatment. Studies on human samples have shown that DNase I treatment of ascites supernatants inhibits NET release by depleting both genomic DNA (gDNA) and mitochondrial DNA (mtDNA) (146). Importantly, exposure to ascites reprogrammed neutrophils toward an immunosuppressive phenotype that inhibited T cell proliferation, suggesting a role for NETosis in tumor-induced immune evasion. In clinical studies, high levels of mitochondrial DNA (mtDNA) and neutrophil elastase—markers of NETosis—in ascites were associated with significantly shorter progression-free survival, indicating that tumor-derived components such as mtDNA can trigger NET formation, platelet activation, and subsequent metastatic spread (146).

#### 4.2.1 NETs as diagnostic and prognostic markers

In addition to their role in OC TME, several studies had proposed the possible diagnostic and prognostic significance of NET markers. In a study of ascites samples, high mtDNA levels correlated with shorter progression-free survival and enhanced NET and platelet activation, suggesting that mtDNA may serve as a prognostic marker and therapeutic target (146). Similarly, Montes et al. (2023) reported that elevated levels of NETosis biomarkers—including cell-free DNA (cfDNA), CitH3, calprotectin, and MPO—were detected compared to controls, suggesting their roles in minimally invasive surrogate biomarkers for HGSOC (128). Furthermore, a prospective two-center study involving 188 patients with newly diagnosed EOC, high pretreatment serum levels of genomic DNA, myeloperoxidase (MPO), and citrullinated histone H3 (CitH3)—markers of neutrophil activation and NET formation—were independently associated with worse overall survival (124). However, in contrast to these findings, Dobilas et al. analyzed plasma samples from 199 women with adnexal masses found no significant differences in circulating NETs markers (H3Cit-DNA and dsDNA) between benign, borderline, and malignant groups, suggesting limited diagnostic value in this context (129). Collectively, these findings suggest that while NET markers hold promise for prognostication in OC, their diagnostic utility remains context-dependent and warrants further investigation.

#### 4.2.2 NETs in OC progression

NETs have been shown to promote tumor progression through direct interactions with cancer cells. In lung carcinoma models, NETs were found to physically bind tumor cells, and this interaction was abolished by DNase or neutrophil elastase inhibitors, suggesting a functional role of NETs in tumor adhesion and spread (147). In OC, the relationship between NETs and disease progression appears complex and, at times, contradictory. Yamamoto et al. reported that OC-induced neutrophilia and elevated G-CSF levels contribute to NET formation, potentially promoting cancer dissemination (148). Similarly, Lee et al. demonstrated that the metastatic tropism of OC is facilitated by NET formation in the premetastatic omental niche, which traps circulating tumor cells and enhances their seeding efficiency (145). However, Muqaku et al. observed a paradoxical association between NET formation and improved overall survival in patients with HGSOC (131).

#### 4.2.3 NETs in OC chemoresistance and metastasis

Recurrence and chemoresistance are the primary causes of mortality in OC. An emerging concept is that NETs may directly impair the efficacy of chemotherapy. Tamura et al. (2022) demonstrated that neutrophils stimulated with PMA or LPS release NETs that physically bind chemotherapy agents, such as doxorubicin (DOX). In 3D culture models of OC, the presence of NETs markedly reduced DOX-induced apoptosis in cancer cells. Mechanistically, DOX was captured by the NET fibers, limiting its bioavailability. Importantly, co-treatment with DNase I dismantled the NET structures and restored DOX cytotoxicity, indicating that NETs may act as drug-absorbing scaffolds within the TME (130). These findings suggest that targeting NETs may represent a promising therapeutic strategy to enhance the efficacy of chemotherapy, particularly in malignancies with NET-rich microenvironments.

Moreover, NETs have been shown to facilitate tumor invasion and metastasis in the TME by inducing tumor‐related inflammatory reactions (149), accelerating EMT, trapping circulating tumor cells, and increasing vascular permeability (150). NETs, stimulated by inflammatory factors secreted by OC, play a critical role in establishing the pre-metastatic omental niche. In murine models, omental colonization was significantly reduced in mice with neutrophil-specific deletion of peptidyl arginine deiminase 4 (PAD4), an enzyme essential for NET formation (145). Similarly, pharmacological inhibition of PAD4 suppressed NET formation and diminished metastatic implantation (145). In a recent study (2025), the same research group further demonstrated that neutrophils infiltrating the omentum in early-stage OC undergo NETosis, depositing NETs that contribute to the recruitment of IL-10–producing innate B cells via NET-induced CXCL13 expression. These B cells subsequently expand local Treg populations through the secretion of IL-10, establishing an immunosuppressive niche that supports tumor cell implantation and proliferation (123). Moreover, a novel study pointed out that upregulating miR142 can dismiss the recruitment of neutrophil with in TME by the expression of CXCL1 regulated by miR-146a, which shed light on a potential therapeutic strategy (151).

Neutropenia, a frequent adverse effect of platinum-taxane chemotherapy, affects approximately 60% of OC patients (152). To mitigate this, G-CSF is commonly administered. However, G-CSF has also been implicated in promoting NET formation, raising concerns about its potential to exacerbate metastasis (145). Notably, G-CSF has been shown to promote N2-type neutrophil polarization in breast cancer (153), further suggesting its potential role in immunosuppressive and tumor-supportive processes. These findings underscore the need for careful evaluation of G-CSF use in patients with a high risk of metastatic spread.

Recent studies have revealed that NETs may reactivate dormant tumor cells, contributing to cancer recurrence and metastasis (154, 155). This process is mediated by NET-associated proteases that remodel the extracellular matrix (ECM), particularly laminin. Laminin degradation exposes new epitopes that activate integrin signaling in dormant cancer cells, triggering their proliferation (155). This highlights a novel and concerning mechanism by which NETs promote tumor relapse.

## 5 TAMs and TANs crosstalk in the TME

Originating from a shared myeloid progenitor lineage, TAMs and TANs play diverse and complementary roles in nearly all stages of tumor development and metastatic progression. Although extensive research effort has been done on neutrophil-lymphoid interactions, fewer studies have examined neutrophil-myeloid cell crosstalk in the OC TME context. Activated neutrophils release IL-8 and TNF-α, which recruit macrophages to the site of inflammation (156). Neutrophils release chemokines such CCL2, CCL3, and CCL4, which draw monocytes and dendritic cells and aid in the recruitment of more myeloid cells into the TME (157). Research has demonstrated that TANs isolated from HCC patients release significant amounts of CCL2 and CCL17, which promote the migration and *in vitro* activation of macrophages and Treg cells in HCC (158, 159).

Expanding on these interactions, Kumar et al. demonstrated that in multiple mouse tumor models, pharmacological inhibition or antibody-mediated neutralization of colony-stimulating factor 1 receptor (CSF1R) led to a compensatory increase in the infiltration of polymorphonuclear myeloid-derived suppressor cells (PMN-MDSCs; CD11b^+^ Ly6C^lo^ Ly6G^+^) (160). These PMN-MDSCs, recruited by carcinoma-associated fibroblasts, ultimately undermined the anticipated therapeutic efficacy of CSF1R blockade (160). Later, in a study by Wang et al., a panel of eight mouse triple-negative breast cancer models was used to demonstrate that tumors did not uniformly recruit TANs and TAMs (161). Despite sharing the same breast cancer subtype, these tumors could be further immunologically subtyped into two distinct subtypes: neutrophil-enriched subtypes (NES, characterized by CD11b+ Ly6Cmid Ly6G+ cells) and macrophage-enriched subtypes (MES, characterized by CD11b+ Ly6G- Ly6C- F4/80+ cells). A mutual exclusivity was observed between TANs and TAMs, whereby the depletion of one population led to the upregulation of the other (161). This reciprocal regulation suggests a complex interplay and potential compensatory dynamics between TANs and TAMs that may influence tumor progression and therapeutic response. Translating these insights to OC, it becomes imperative to further elucidate the mechanisms governing TAN-TAM crosstalk to develop more effective combinatorial therapeutic strategies.

The investigations collectively demonstrated intricate crosstalk between TANs, TAMs, and other components of the TME, highlighting the necessity for integrated therapeutic approaches that consider the plasticity and compensatory pathways within the myeloid cell network.

## 6 Treatment targeting macrophages and neutrophils in OC

OC has a poor response to immune checkpoint inhibitors, which is largely due to robust immunosuppressive TME and poor T cell immunity. The immunosuppressive TME in OC is predominantly driven by TAMs with tumor-promoting properties. The heterogeneity of immune cell populations within the TME poses significant challenges for developing effective therapies for OC. To overcome these challenges, current treatment strategies increasingly focus on personalized approaches that target the unique immune landscape of each tumor.

Growing information from preclinical and clinical investigations has deepened our understanding of the critical role TAMs play in driving tumor progression and resistance to therapies. As a result, TAMs have emerged as a key target in the development of novel cancer therapies aiming at improving outcomes for patients with OC. The most extensively studied strategies for targeting TAMs are: (1) inhibiting recruitment to the TME, (2) depleting TAM populations or disrupting their survival, (3) reprogramming or repolarizing toward an anti-tumor phenotype, (4) restoring their innate tumor-suppressive functions, (5) suppressing tumor-promoting activities, and (6) CAR-macrophages (CAR-Ms) (32, 162). However, TAM-targeting therapies face challenges due to TAMs’ high plasticity and heterogeneity, with their diverse phenotypes varying by tumor type and location within the same tumor (163). Recently, Klichinsky et al. pioneered the generation of CAR-Ms, macrophages that show antigen-specific phagocytosis and tumor clearance *in vitro*, demonstrating their ability to target tumor cells and activate adaptive immunity in humanized mice (164). CAR-Ms targeting HER2 and CD47 displayed antigen-specific phagocytosis of OC cells *in vitro* and the ability to activate CD8+ cytotoxic T lymphocytes (165). On the other hand, selective elimination of FRβ+ TAMs (M2-like) via CAR-T cells reshapes the TME, leading to improved antitumor immunity and tumor-directed CAR-T therapies (166).

Targeting NETs represents a promising strategy for boosting the immune response against tumors and improving the efficacy of existing cancer treatments; nevertheless, this concept shall be assessed rigorously in clinical studies (167). Currently, most therapeutic studies were performed on animal models (167). For example, treatment with NET-inhibiting agents reduced omental colonization in NET-competent mice without affecting neutrophil influx. In neutrophil-depleted mice, omental metastasis was inhibited by about 70%, indicating that NET formation plays a key role in tumor development (145). Biomarkers associated with NETs formation, such as H3Cit and MPO-DNA, may have prognostic significance for cancer patients (168). Understanding the role of NETs in the TME of OC is critical for developing targeted therapies, ultimately improving patient outcomes and facilitating personalized treatment approaches.

## 7 Conclusion

OC is shaped by a profoundly immunosuppressive tumor microenvironment, in which TAMs and TANs exert decisive, yet dynamic, influences on disease progression. Both cell types demonstrate functional plasticity, contributing to immune evasion, angiogenesis, and resistance to therapy. The development of NETs provides an additional layer of immunomodulation and metastatic potential. Recent research on the reciprocal regulation of TAMs and TANs has revealed compensatory mechanisms that may undermine the efficacy of monotherapy targeting either cell population alone. These findings underscore the need for combinatorial strategies that consider the broader myeloid landscape. Novel approaches, such as CAR-engineered macrophages and NET inhibition, offer promise but require further validation in clinical settings. A deeper understanding of the spatial and functional dynamics of innate immune cells in the OC TME is essential. Future efforts should focus on identifying predictive biomarkers and developing rational, immune-targeted therapies that exploit the full potential of myeloid modulation to improve patient outcomes.
